# Data Privacy, Ownership, and Secondary Use of Clinical Data Generated by Continuous Glucose Monitors and Mobile Health Applications: A Review

**DOI:** 10.1177/19322968261455365

**Published:** 2026-06-07

**Authors:** Paulo Dario

**Affiliations:** 1 Instituto Nacional de Saúde Doutor Ricardo Jorge (INSA), Departamento de Promoção da Saúde e Prevenção de Doenças Não Transmissíveis, Lisboa, Portugal

**Keywords:** continuous glucose monitoring, data ownership, data privacy, mHealth, regulatory frameworks, secondary use

## Abstract

The growing use of continuous glucose monitors (CGMs) and mobile health (mHealth) applications has changed how diabetes is managed, allowing real-time tracking of glycemic patterns and remote clinical decision-making. These technologies also generate large volumes of sensitive health data, raising questions about who owns this information, how it is protected, and under what conditions it may be repurposed for research or commercial objectives. This review examines the regulatory frameworks governing CGM and mHealth data in major jurisdictions, with particular attention to the Health Insurance Portability and Accountability Act (HIPAA) in the United States and the General Data Protection Regulation (GDPR) in the European Union. Significant regulatory gaps exist, particularly for consumer-grade devices and direct-to-consumer mHealth applications that fall outside traditional healthcare data-protection frameworks. Data ownership remains legally ambiguous in most jurisdictions, with patients, healthcare providers, device manufacturers, and app developers each holding competing claims. The secondary use of clinical data for research, while it could materially advance diabetes care, raises ethical concerns around informed consent, data de-identification, and the boundaries between clinical care and commercial exploitation. Emerging approaches, including the European Health Data Space, federated learning, and differential privacy, may help balance data utility with individual rights. The review recommends changes to regulation, industry practice, and consent models aimed at reconciling data-driven diabetes research with patient autonomy and privacy.

## Introduction

Continuous glucose monitors (CGMs) sample interstitial glucose every 1-5 minutes, generating up to 1440 readings per day.^1^ Integrated with mobile health (mHealth) applications, insulin pumps, and cloud analytics, they produce a continuous stream that also captures activity, diet, medication, sleep, and geolocation.^2,3^ Time in range and the ambulatory glucose profile are now consensus elements of diabetes care.^4,5^ Recent Food and Drug Administration (FDA) clearance of over-the-counter CGMs,^6^ in a market projected to exceed USD 49 billion by 2033,^7^ indicates adoption beyond clinically diagnosed users.

**Figure 1.**
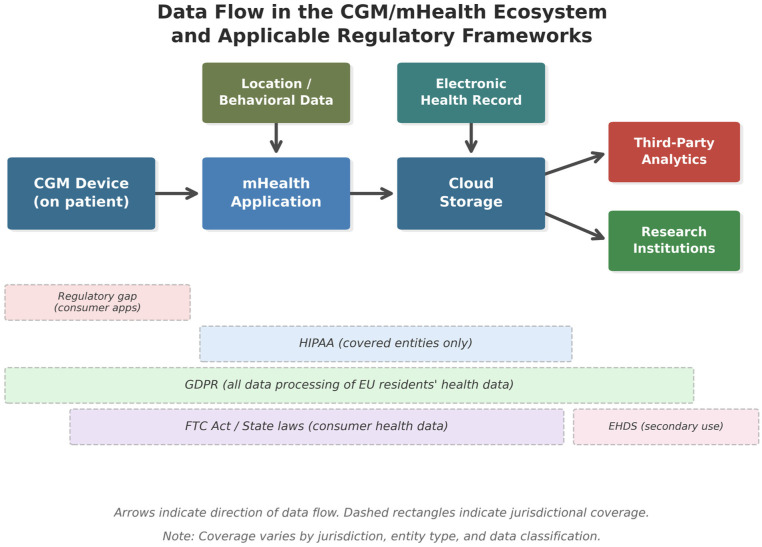
Data flow from CGM devices through mHealth applications to cloud storage, third-party analytics, and research institutions, with applicable regulatory frameworks. Dashed rectangles indicate jurisdictional coverage. Health Insurance Portability and Accountability Act’s (HIPAA’s) entity-based scope leaves a regulatory gap for consumer-grade applications. The GDPR covers all processing of European Union residents’ health data regardless of entity type. The EHDS governs secondary use through Health Data Access Bodies (full implementation expected by 2029).

Yet this same granularity reveals information far beyond glucose. A CGM trace shows meal times, stress responses, sleep patterns, and alcohol consumption.^8^ Combined with smartphone location data, the resulting dataset constitutes a detailed behavioral profile. The data originate from a medical device on the patient’s body, travel through a consumer application, sit on commercial cloud servers, and reach third parties with no connection to the patient’s care.^9^ Figure 1 illustrates this data flow.

Three interrelated problems define this area. First, data-protection regulations contain gaps that leave substantial categories of diabetes device data without clear legal safeguards. Second, the question of who owns patient-generated health data (PGHD) remains unresolved, with patients, clinicians, device manufacturers, and software developers each asserting competing claims.^10^ Third, when researchers wish to repurpose these data, the consent frameworks in place often fail to explain what will happen to the data, who will access them, or for what purpose.^11^ These three problems are usually treated as separate domains; this review treats them together because each is logically dependent on the other two.

This review examines these three problems in the context of CGM and mHealth technologies for diabetes, surveys the regulatory frameworks in the United States (US) and the European Union (EU), and evaluates emerging technical and governance solutions that may help balance data-driven research against the protection of individual rights.

## Data Privacy: Regulatory Frameworks and Their Limitations

### The US Regulatory Environment

In the US, the Health Insurance Portability and Accountability Act (HIPAA) provides the primary legal framework for protecting health information. It applies to healthcare providers, health plans, healthcare clearinghouses, and their business associates.^12^ The Privacy Rule governs how protected health information may be used and disclosed, while the Security Rule establishes technical safeguards for electronic health data.

However, HIPAA’s applicability to CGM and mHealth data is limited by its entity-based rather than data-based scope. Consumer health applications and wearable device manufacturers are generally not covered entities, meaning that glucose data transmitted from a CGM to a manufacturer’s cloud platform, or shared with a third-party wellness application, may fall entirely outside HIPAA’s protections.^13,14^ The Federal Trade Commission (FTC) has tried to fill some of this gap through enforcement actions under Section 5 of the FTC Act and an updated Health Breach Notification Rule (finalized in 2024) that explicitly includes health apps and connected devices.^15^ Some states have gone further. Washington’s My Health My Data Act (2023), for instance, creates a private right of action that allows individuals to sue companies that collect or share their consumer health data without authorization.^16^ However, My Health My Data Act (MHMDA) leaves the rules on permitted downstream uses of collected data incompletely defined; in practice, users are often required to grant broad data-use permissions to activate their CGM, which makes the consent obtained less meaningful than the statute implies. Connecticut, Nevada, and other states have introduced similar legislation, creating a trend toward state-level regulation that may produce a patchwork of obligations for CGM and mHealth companies.

Despite these developments, the US regulatory environment remains fragmented. A CGM user’s glucose data may be governed by HIPAA when accessed by a clinician through an electronic health record, by the FTC Act when processed by the device manufacturer’s application, and by state consumer protection laws when shared with third-party analytics services, or it may fall into a regulatory gap where none of these frameworks apply.^9,13^ A practical consequence is that CGM users cannot reliably anticipate which legal regime will govern any given use of their data, weakening informed consent.

### The EU Regulatory Framework

The EU’s General Data Protection Regulation (GDPR), in force since 2018, takes a fundamentally different approach by regulating data processing on the basis of the data itself rather than the entity processing it. Under GDPR, health data are classified as a special category of personal data subject to heightened protections, regardless of whether the data controller is a healthcare provider, a device manufacturer, or an application developer.^17^ Data subjects hold enforceable rights of access, rectification, erasure, and data portability. Processing of health data generally requires explicit consent or another specified legal basis.

The Medical Devices Regulation, applicable since May 2021, imposes additional requirements on devices that generate health data, including software for clinical decision support.^18^ Limited notified-body capacity has slowed enforcement, and transitional provisions extend MDR requirements to devices already on the market. Compliance costs also deter small and mid-sized firms from market entry. One recent analysis of 11 diabetes-management devices and apps marketed in Europe found that only 3 were registered in EUDAMED, and the privacy policies varied markedly in how transparent they were about data sharing.^19^ Of the 11 devices analyzed, a majority sent data to third parties for purposes unrelated to the user’s care, and exercising GDPR rights often proved technically difficult.

### Cross-Jurisdictional Challenges

The global nature of diabetes device networks creates additional complexities. A CGM manufactured in the US, used by a patient in the EU, and transmitting data to servers in a third jurisdiction is subject to multiple overlapping regulatory regimes. Data transfer mechanisms such as Standard Contractual Clauses and adequacy decisions under GDPR impose constraints on cross-border data flows, but enforcement remains inconsistent.^20^ Interoperability limitations compound this problem: A narrative commentary from EU, UK, and US perspectives documented how proprietary ecosystems and incompatible data formats impede cross-platform data sharing.^21^ For multinational clinical trials and registry studies aggregating CGM data across borders, these jurisdictional and technical barriers are practical obstacles to the secondary use of data for research.^22^ Table 1 compares these frameworks along scope, patient rights, coverage, and key limitations.

**Table 1.** Comparison of Regulatory Frameworks Governing CGM and mHealth Data.

Dimension	HIPAA (US)	FTC/State Laws (US)	GDPR (EU)	EHDS (EU)
Scope	Covered entities and business associates only	Consumer apps and devices not covered by HIPAA	All processing of EU residents’ personal data	Secondary use of health data across EU member states
Data types	Protected health information (PHI)	Consumer health data (varies by state)	All health data (special category)	Electronic health data including device-generated data
Patient rights	Access and copy; no ownership right	Varies; WA MHMDA: consent required, private right of action	Access, rectification, erasure, portability, withdrawal of consent	Primary use: access, portability. Secondary: opt-out for certain uses
CGM/mHealth coverage	Limited: consumer devices typically excluded	Partial: FTC enforces for non-HIPAA health apps	Comprehensive: applies to all data controllers	Comprehensive: includes device data in EHR and registries
Enforcement	OCR; civil/criminal penalties	FTC enforcement; state attorneys general; private actions (WA)	National DPAs; fines up to 4% global turnover	Health Data Access Bodies; penalties per member state law
Key limitation	Entity-based: consumer devices fall outside scope	Fragmented patchwork; inconsistent across states	Enforcement inconsistency across member states; portability limited in practice	Full implementation not expected until 2029

Abbreviations: CGM, continuous glucose monitor; DPA, data-protection authority; EHDS, European Health Data Space; EHR, electronic health record; FTC, Federal Trade Commission; GDPR, General Data Protection Regulation; HIPAA, Health Insurance Portability and Accountability Act; mHealth, mobile health; OCR, Office for Civil Rights; WA MHMDA, Washington My Health My Data Act.

The cybersecurity of connected diabetes devices adds a further dimension. Insulin pumps and CGMs that communicate wirelessly are potentially vulnerable to data interception and, in a worst-case scenario, manipulation of dosing commands. The Diabetes Technology Society Cybersecurity Standard for Connected Diabetes Devices (DTSec) and IEEE 2621 standards provide frameworks for wireless diabetes device security,^23^ but adoption is uneven, and mandatory cybersecurity certification for diabetes devices transmitting health data remains absent in both the US and the EU.

## Data Ownership: Competing Claims and Legal Ambiguity

The question of who owns health data generated by CGMs and mHealth applications has no clear legal answer in most jurisdictions. Unlike physical property, data do not fit neatly into existing ownership frameworks, and the concept of data ownership itself is contested among legal scholars.^24,25^ The need for clear medical data ownership laws has been articulated as a prerequisite for both translational research and patient empowerment.^26^

In the US, healthcare providers own the physical medical record, while patients have a right of access to the information within it. HIPAA grants patients the right to request, view, and obtain copies of their health information, but this right of access does not equate to ownership.^12^ For data generated by consumer CGMs and mHealth applications, control over data is typically governed by terms of service agreed to by the user at device activation or application download. Analyses of mHealth data-sharing practices have shown that health-related apps routinely transmit user data to third-party services, often for purposes unrelated to clinical care.^27^ Studies of digital service agreements suggest that up to 97% of users accept such terms without reading them, transferring control of their data through a mechanism that does not reflect genuine informed decision-making.^28^

The GDPR reframes the problem. Rather than establishing data ownership in a proprietary sense, the GDPR articulates a rights-based framework centered on the data subject. Individuals hold enforceable rights over the processing of their data, including the right to withdraw consent, the right to erasure, and the right to data portability.^17^ The right to portability has been proposed as a mechanism to give patients with diabetes greater control over their CGM data by enabling them to transfer their data between devices and platforms.^29^ In practice, however, interoperability barriers and proprietary data formats have limited the exercise of this right. Open-source initiatives such as Tidepool, which aims to standardize diabetes device data formats and enable cross-platform access,^30^ aim to overcome these barriers, but adoption remains limited. Device manufacturers have little economic incentive to facilitate data portability, as data lock-in functions as a competitive advantage.^19,31^ User experience studies have confirmed that vendor lock-in remains a tangible barrier for patients attempting to access or transfer their own diabetes data.^32^

The emergence of data cooperatives and patient-led data trusts offers an alternative governance model in which individuals collectively manage access to their health data.^33^ Patients retain decision-making authority over data use, and any benefits from sharing are distributed according to collectively agreed terms. While nascent, these models address the power asymmetry between individual patients and device manufacturers.^34^

There is also the question of derived data. Machine learning models trained on aggregated CGM readings generate predictions, risk scores, and treatment recommendations that are not the original data—they are something new. These derived insights can have commercial value independent of any one patient’s contribution, yet regulatory frameworks do not address who owns or controls them.^35^ The potential for commercial exploitation is real, as mHealth data-sharing practices document routine transmission of user data to unrelated third parties.^27,36^ This is the central unresolved problem in current diabetes-data governance: Ownership rules designed for raw clinical records do not extend to the algorithmic products derived from them.

## Secondary Use of Clinical Data for Research

### The Promise

The secondary use of CGM and mHealth data for research could yield practical benefits. Large-scale CGM datasets have enabled validation of time in range as a meaningful clinical trial outcome measure and established associations between CGM-derived metrics and long-term complications such as diabetic retinopathy.^37,38^ Registry studies such as the Swedish National Diabetes Register and the T1D Exchange have demonstrated the value of aggregated clinical data in complementing randomized controlled trials.^39,40^ Data-driven approaches such as cluster analysis identifying distinct subgroups of adult-onset diabetes with differing complication profiles^41^ suggest that CGM-derived data could contribute to more individualized management.

### Ethical Concerns

Secondary use of data raises 3 ethical concerns that are specific to the diabetes-technology context. The first relates to consent. Most CGM users provide consent for data collection in the context of their clinical care or device use, but the scope of this consent with respect to future research applications is often ambiguous. Broad consent models, which authorize unspecified future uses of data, have been criticized for failing to meet the standard of informed consent, particularly when the future uses may include commercial research by the device manufacturer or its partners.^42,43^ Dynamic consent platforms, which allow individuals to make ongoing decisions about how their data are used, have been proposed as an alternative, though their practical implementation at scale remains limited.^44^ Qualitative evidence suggests that adults with type 1 diabetes already exercise selective data sharing: Some choose not to use CGM remote-monitoring features due to concerns about anxiety, loss of independence, and insufficient customization of sharing options.^45^

The second concern relates to deidentification. CGM data present particular challenges for anonymization due to their temporal density and potential linkage with behavioral and location data. A single CGM trace generates a temporally dense physiological signature that may suffice to reidentify an individual even after direct identifiers are removed. Time-series health data have been shown to be reidentifiable with high accuracy through pattern matching.^46,47^ A 2023 systematic review specifically examining deidentification of wearable device data concluded that current anonymization methods may give a false sense of security, as the temporal granularity of sensor-generated data creates unique individual signatures resistant to standard deidentification approaches.^48^ The HIPAA Safe Harbor standard, which specifies 18 types of identifiers to be removed, was not designed with continuous physiological monitoring data in mind, and its adequacy for CGM datasets is questionable. A 2025 analysis of privacy policies across 17 leading wearable manufacturers found that 76% received high-risk ratings for transparency and that the majority shared data with third parties under terms that users were unlikely to understand.^49^ The implication is that ‘deidentified’ CGM datasets should be treated as identifiable for governance purposes; the question is no longer whether reidentification is possible, but how to manage the risk.

The third concern involves the boundary between research and commercial exploitation. Device manufacturers that collect CGM data for product improvement may also use those data, or insights derived from them, for commercial applications such as development of proprietary algorithms or sale of aggregated analytics to pharmaceutical companies and insurers.^27,36^ When research and commercial objectives converge within the same data environment, the distinction between use that benefits the patient community and use that primarily serves corporate interests becomes difficult to maintain.

### The European Health Data Space

The European Health Data Space (EHDS) regulation, published in the Official Journal of the EU on March 5, 2025, is the most detailed legislative effort to date to create a governance framework for secondary use of health data.^50^ The EHDS establishes Health Data Access Bodies in each member state to evaluate and authorize requests for secondary use of health data, including scientific research and public health surveillance. Data access must occur through secure processing environments, and certain uses, including insurance risk assessment and direct marketing, are prohibited.^51^

For diabetes research, the EHDS could facilitate cross-border studies using CGM and mHealth data while maintaining consistent governance standards across member states. However, implementation challenges remain substantial, including the need to harmonize data formats across different CGM platforms, establish interoperability standards for mHealth applications, and build the institutional infrastructure required to operate Health Data Access Bodies effectively in each member state. Efforts such as the iCoDE-2 standards project, which aims to integrate connected diabetes device data into electronic health records through standardized formats, illustrate both the potential and the complexity of achieving interoperability in this domain.^52^ The timeline for implementation is phased: While the regulation entered into force on March 26, 2025, the full application of secondary use provisions is not expected until 2029, leaving a multi-year gap during which current fragmented approaches will continue to govern research access to diabetes device data.^50,53^

## Emerging Technical Solutions

Technical approaches to privacy preservation offer a complementary path to regulatory reform. Federated learning, in which machine learning models are trained on decentralized data without the data leaving the local institution, has been applied to diabetes prediction and classification tasks with results comparable to centralized approaches.^54,55^ Differential privacy, which introduces calibrated noise into datasets or model outputs to prevent the identification of individual records, has been combined with federated learning in diabetes-specific implementations.^56^ Homomorphic encryption, which allows computation on encrypted data, and synthetic data generation, which creates artificial datasets preserving the statistical properties of the original, are additional privacy-enhancing technologies (PETs) that may enable research on sensitive CGM data without exposing individual records.^57^

These technical solutions have important limitations. Federated learning introduces communication overhead and may produce models biased toward institutions with larger datasets. Differential privacy involves an inherent trade-off between privacy protection and data utility, and the appropriate calibration of noise parameters for glucose data has not been established. Synthetic data may fail to capture rare but clinically relevant patterns, such as nocturnal hypoglycemia episodes. No technical solution eliminates the need for appropriate governance and consent frameworks; PETs should be understood as tools that expand the range of options available within a broader governance structure.^58,59^

Blockchain-based approaches to consent management and data provenance tracking have also attracted interest in this area. By recording data access events on a distributed ledger, blockchain systems could provide patients with an auditable record of who has accessed their CGM data, when, and for what stated purpose. Proof-of-concept implementations have been described, though scalability concerns, energy consumption, and the tension between immutable records and the GDPR right to erasure remain barriers that require careful architectural design.^35,57^

## Conclusions

The rapid adoption of CGMs and mHealth applications in diabetes management has created a data environment that outpaces the regulatory, legal, and ethical frameworks designed to govern it. The data produced by these devices are simultaneously clinical records, consumer data, and commercial assets, and the boundaries between these categories are poorly defined by existing law. Privacy protections remain fragmented, with significant gaps for consumer-grade devices that fall outside HIPAA and face inconsistent enforcement under GDPR. Data ownership remains legally ambiguous, and current consent mechanisms fail to provide patients with meaningful control over how their data are used. This review focuses on the US and EU regulatory environments; other major diabetes populations, including those in China, India, and Brazil, face distinct regulatory challenges that warrant separate analysis.

Secondary use of diabetes device data for research can improve clinical outcomes, but not responsibly without addressing ethical concerns around consent, de-identification, and the conflation of research with commercial interests. The EHDS is the largest effort to date to create a coherent governance framework for secondary use of health data, though its effectiveness will depend on how well member states implement it and whether they invest in the required institutional infrastructure.

Five actions follow from this analysis and are summarized in Table 2.

**Table 2.** Recommended Actions to Address Privacy, Ownership, and Secondary-Use Gaps in CGM and mHealth data.

#	Stakeholder	Recommended action	Specific implementation step
1	US Congress and EU institutions	Extend health-data protections to all entities processing medical-device data, irrespective of covered-entity status	Amend HIPAA (or enact a federal equivalent) to define protected health information by data type rather than by entity; align GDPR enforcement to consumer-grade CGM and mHealth platforms
2	Device manufacturers and software developers	Adopt interoperability and data-portability standards as a release-blocking requirement	Conform to iCoDE-2 specifications for EHR integration and to Tidepool open-source data formats; provide standardized export and portability APIs at product approval
3	Researchers, IRBs, and EHDS Health Data Access Bodies	Make privacy-enhancing technologies the default for secondary analysis of CGM data	Require federated learning, differential privacy, or equivalent PETs in the standard IRB or HDAB review checklist; reject protocols that centralize raw CGM records without explicit justification
4	Funders, sponsors, and IRBs	Replace static broad consent with dynamic consent for connected diabetes-technology studies	Mandate dynamic consent platforms in protocols using CGM or mHealth data, allowing participants to grant, modify, or withdraw permission per use category over time
5	Regulators and AID-system manufacturers	Develop privacy and cybersecurity rules specific to closed-loop automated insulin-delivery systems	Issue AID-specific guidance covering real-time CGM, controller, and pump data flows; require certification under DTSec or IEEE 2621 (or equivalent) before market authorization

Abbreviations: AID, automated insulin delivery; API, application programming interface; CGM, continuous glucose monitor; DTSec, Diabetes Technology Society Cybersecurity Standard for Connected Diabetes Devices; EHDS, European Health Data Space; EHR, electronic health record; GDPR, General Data Protection Regulation; HDAB, Health Data Access Body; HIPAA, Health Insurance Portability and Accountability Act; IEEE 2621, IEEE Standard for Wireless Diabetes Device Security; IRB, institutional review board; mHealth, mobile health; PET, privacy-enhancing technology.

The people who wear these devices and generate these data need a framework that protects their privacy and autonomy without blocking the research that may improve their care. Without one, the default is an unregulated environment in which commercial interests set the terms of access and use, and in which the trust that sustains both clinical adoption and research participation steadily erodes.
